# Removal and Oxidation of Low Concentration *tert*-Butanol from Potable Water using Nonthermal Plasma Coupled with Metal Oxide Adsorption

**DOI:** 10.1021/acsestengg.4c00166

**Published:** 2024-08-20

**Authors:** Cristina E. Stere, Maicon Delarmelina, Mbongiseni W. Dlamini, Sarayute Chansai, Philip R. Davies, Graham J. Hutchings, C. Richard A. Catlow, Christopher Hardacre

**Affiliations:** † Department of Chemical Engineering, University of Manchester, Oxford Road, Manchester M13 9PL, U.K.; ‡ Cardiff Catalysis Institute, School of Chemistry, Cardiff University, Cardiff CF10 3AT, U.K.; § Max Planck-Cardiff Centre on the Fundamentals of Heterogeneous Catalysis FUNCAT, Cardiff Catalysis Institute, School of Chemistry, Cardiff University, Main Building, Park Place, Cardiff CF10 3AT, U.K.; ∥ Department of Chemistry, University College London, 20 Gordon St., London WC1 HOAJ, U.K.

**Keywords:** *tert*-butanol, Al_2_O_3_, plasma DBD, adsorption, water treatment

## Abstract

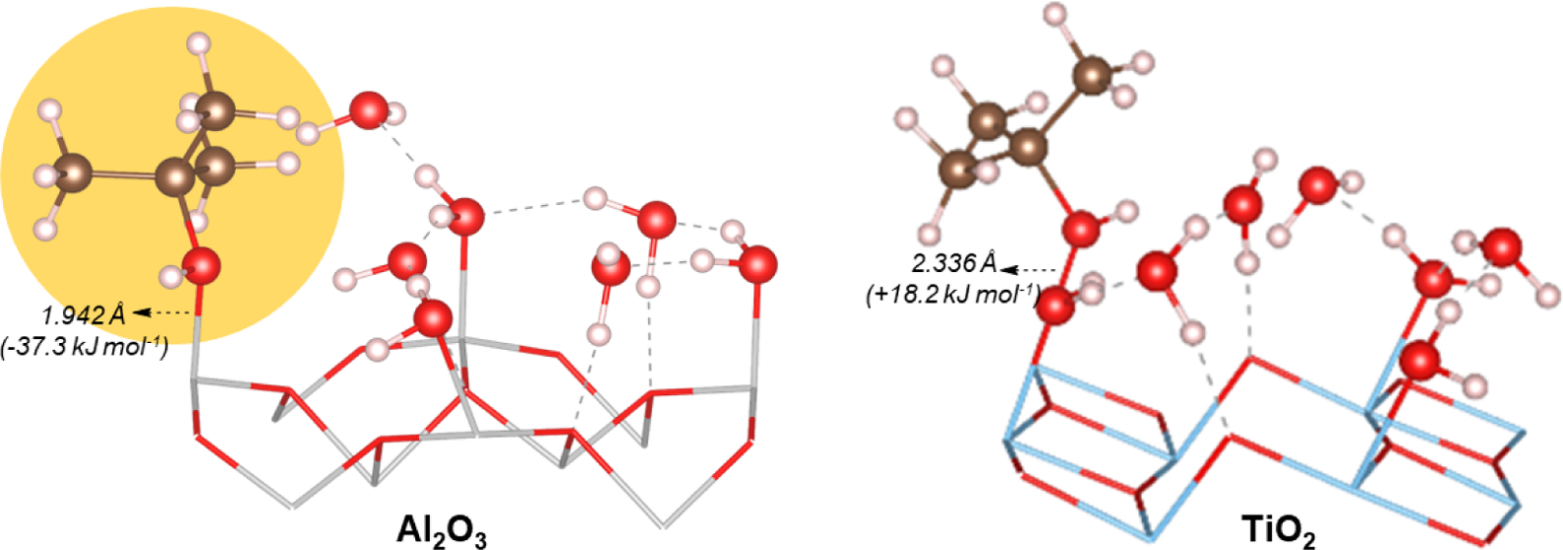

Taste and odor are crucial factors in evaluating the quality of drinking water for consumers. Geosmin is an example of a pollutant commonly found in potable water responsible for earthy and musty taste, and odor even at low concentrations. We have investigated the use of a hybrid two-step adsorption-mineralization process for low-level volatile organic compounds removal from potable water using dielectric barrier discharge over common metal oxides (MO). The system proposed is a proof of principle with *tert*-butanol (TBA) used as a model compound for geosmin removal/degradation during wastewater treatment when combined with an appropriate metal oxide adsorbent. Initial assessments of the adsorption properties of titania by density functional theory (DFT) calculations and experimental tests indicated that the adsorption of geosmin and TBA with water present results in only weak interactions between the sorbate and the metal oxide. In contrast, the DFT results show that alumina could be a suitable adsorbent for these tertiary alcohols and were reinforced by experimental studies. We find that while there is a competitive effect between the water and TBA adsorption from gaseous/liquid feed, the VOC can be removed, and the alumina will be regenerated by the reactive oxygen species (ROS) produced by a dielectric barrier discharge (DBD). The use of alumina in conjunction with NTP leads to efficient degradation of the adsorbate and the formation of oxygenated intermediates (formates, carbonates, and carboxylate-type species), which could then be mineralized for the regeneration of the adsorbent. A reaction mechanism has been proposed based on the *in-situ* infrared measurements and DFT calculations, while the removal of TBA with conventional heating is indicative of a gradual desorption process as a function of temperature rather than the destruction of the adsorbate. Furthermore, steady performance was observed after several adsorption–regeneration cycles, indicating no alteration of the adsorption properties of alumina during the NTP treatment and demonstrating the potential of the approach to be applied in the treatment of high throughput of water, without the challenges faced by the biocatalysts or formation of toxic byproducts.

## 1 Introduction

The quality of potable water is of paramount importance for society, with a global water treatment systems market estimated to be USD 23.1 billion in 2022. Moreover, the United Nations recognized access to clean water as a sustainable development goal.^1^ In this context, taste and odor represent critical criteria in the evaluation of potable water quality for consumers.

Two common contaminants responsible for the most unpleasant and widely distributed off-flavors in freshwater bodies are trans-1,10-dimethyl-trans-9-decalol (geosmin) and 2-methylisoborneol (MIB). They are secondary metabolites produced by actinomycetes (bacteria) and blue-green algae (cyanobacteria), which are easily identified in many sources of drinking water because of their earthly/musty taste and smell, even in concentrations below 10 ng L^–1^. The presence of geosmin and MIB in water is often perceived by consumers as an indication of poor water quality despite the very low concentration levels. Thus, while they are not necessarily posing a health hazard, the economic impact on the potable water sector is high.

The removal of volatile organic compounds (VOCs) from water commonly involves adsorption on activated carbon, biocatalysis, or advanced oxidation processes (AOPs), such as H_2_O_2_/O_3_ process, ultraviolet (UV)/H_2_O_2_ process, UV/O_3_ process, photo-Fenton reaction, and titanium dioxide (TiO_2_)-assisted photocatalytic process.^2−8^ AOPs for water treatment have been studied extensively,^9−16^ and particularly, the use of plasma in direct contact with water^15,17−21^ has been regarded as a promising technology for water treatment. Despite the wide utilization of these processes in the water treatment industry, there are still significant issues to be addressed, such as the implementation of additional removal steps, recyclability issues or byproduct formation.^12,15,18,22−28^

This study focuses on combining the advantages of two processes commonly used in water treatment: the adsorption of VOC on metal oxides (MO) followed by mineralization of the sorbate with the regeneration of the sorbent materials using dielectric barrier discharge (DBD) as an AOP. The aim of such a treatment of the VOCs is to use low-cost, commercially available materials for capturing geosmin and MIB from potable water and subsequently to decompose these organic compounds into CO_2_ gas (referred to as mineralization), without affecting the MO adsorptive properties. Mineralisation of the adsorbates would, in this case, be a critical aspect to consider, which is normally achieved via oxidation, either chemically or photochemically. While plasma alone, in an oxidative environment, is a powerful AOP due to the formation of atomic oxygen, hydroxyl radicals (HȮ), ozone as well as radical ions, namely O_2_˙^+^, O_2_˙^–^, O_3_˙^–^, highly energetic free electrons (e^–^), intense electric field, UV radiation, etc.,^29^ it can also lead to harmful byproducts formation, as it is a nonselective process. Thus, the combination of NTP with a good adsorbent could be an efficient process for degrading VOC from water. Metal oxides^9^ (titanium dioxides, cerium oxides, aluminum oxides, zirconium oxides, iron oxides, manganese oxides, zinc oxides, etc.) can be used as VOC sorbents due to their high adsorption capacity, low-cost, simplicity of separation, good stability, photocatalytic, antibacterial, and antifungal activity, and they have also proved to be suitable materials for a number of plasma-catalysis applications, including VOC oxidation, nitric oxide decomposition, water gas shift (WGS), CO_2_ hydrogenation and reforming, as well as water treatment.^10,13,30−43^ Several studies have shown good conversions on applying corona discharge, gliding arc,^44^ as well as DBD^45^ pulsed discharge plasma for water decontamination.^46,47^

For this study, titania and alumina have been chosen as testing materials for their reported use in AOPs,^9,17,33,48−50^ such as photocatalysis and plasma, respectively, and the plasma reactor chosen was a DBD for the simplistic design, ease of implementation on an industrial scale, and ease of incorporation of the catalytic materials. Furthermore, the dielectric barrier is important as it prevents arc formation and permits the generation of cold microdischarges on the surface of the adsorbent. For ease of understanding and initial assessment of the process, the simplest tertiary alcohol, *tert*-butanol (TBA), was used as a model compound for two of the most problematic biogenic odor compounds reported for drinking water, the tertiary alcohols geosmin, and MIB.^20,51−53^ These alcohols raise similar issues for the removal/treatment from water consisting of extreme stability, low Henry’s law constant, resistance to natural degradation, boiling, and conventional treatment processes. The existing literature indicates that the critical pathways and initial steps of geosmin and MIB degradation using advanced oxidation processes involve the degradation, oxidation, bond cleavage, and demethylation of the tertiary alcohol group.^54,55^ There is a cascade of secondary reactions that can lead to carbon dioxide and water, but it is the reactivity of the tertiary carbon attached to the OH group that is important. TBA contains a structurally analogous group to both MIB and geosmin and, therefore, provides similar reactivity. It is also important to note that TBA is also a significant pollutant as a result of leaking underground storage tanks or from ethyl *tert*-butyl ether (ETBE) and methyl *tert*-butyl ether (MTBE) contamination, as it does not readily adsorb on suspended sediments/solids in water and its biodegradation can take weeks to months.^56^ Moreover, TBA is expected to have very high mobility based upon a reported soil organic carbon–water partition coefficient (Koc) of 37 if released to soil.^57^ Thus, the system proposed here is a proof of principle for a hybrid two-step adsorption-mineralization process over metal oxides, using TBA as a model molecule for tertiary alcohol-VOCs from water and DBD as AOP.

## 2 Experimental Section

The materials used in this study as adsorbents were γ-Al_2_O_3_ obtained from AlfaAesar (BET surface area of 146 m^2^ g^–1^) and TiO_2_ P90 from Aeroxide (12.8% rutile and 87.2% anatase, BET surface area of 103 m^2^ g^–1^).

For proof of principle, the initial tests were carried out in a diluted gas feed, using a DBD reactor. The gases for the reaction mixture were supplied by BOC, i.e., 5% Kr/Ar (99.99%), O_2_ (99.99%), and Ar (99.999%), and each gas flow was individually controlled by a Bronkhorst El-Flow mass flow controller. The *tert*-butanol (Sigma–Aldrich) and water vapor were introduced by passing Ar as a carrier gas through separate custom-made saturators. The temperature of the saturators was controlled using a GrantTM GD120 thermostatic bath. The inlet and outlet gas lines were heat traced and maintained at 100 °C to prevent condensation.

### 2.1 Computational Methods

All calculations were performed using the Vienna *ab initio* simulation package (VASP)^58−61^ within the framework of periodic density functional theory (DFT). The electronic structure of all of the systems modeled employed the RPBE^62^ functional combined with Grimme’s semiclassical D3 dispersion correction.^63,64^ Additionally, Hubbard *U* correction was used for all calculations involving TiO_2_ (*U*_Ti(*d*)_ = 4.5 eV), in accordance with a preliminary evaluation.^65^ The electron–ionic core interaction was represented by the projector-augmented-wave (PAW) potentials.^63,64^ Brillouin zone sampling of TiO_2_ was performed using the Monkhorst–Pack scheme with a k-point grid of 7 × 7 × 1 and cutoff energy was set to 550 eV.^65^ After extensive benchmarking of k-point grids and cutoff energies, the most appropriate values for Al_2_O_3_ were defined as those after the energy conversion of the system, i.e., 5 × 5 × 1 and 550 eV (Figure S1, ESI). Gaussian smearing broadening of 0.02 eV was used in all calculations. The Ti 3d^3^4s^1^, Al 3s^2^3p^1^, and O 2s^2^2p^4^ orbitals were explicitly included as valence electrons. Forces and electronic SCF convergence were set at 10^–2^ eV Å^–1^ and 10^–5^ eV, respectively. Dipole corrections were additionally used during all calculations, according to the method by Makov^66^ and others.^67^ The optimized lattice constants obtained at this theory level were used in this work to construct the surface models investigated.

The slab model for the anatase (101) surface (a-TiO_2_ (101)) used a 2 × 1 × 3 supercell containing 24 titanium and 48 oxygen atoms, in which the 8 top titanium and 20 oxygen ions were allowed to relax. For the α-Al_2_O_3_ (0001) surface, a 2 × 2 × 1 supercell containing 48 aluminum and 72 oxygen atoms was employed, in which the 28 top aluminum and 36 oxygen ions were allowed to relax (Figure S1). A vacuum gap of 15 Å in the z direction was added to the surface in order to avoid undesired interactions with the slab images.

All reported adsorption energies (*E*_ads_) were calculated using eq 1, where *E*_(Clean Surface)_ is the total energy of the clean surface, *E*_(Adsorbate)_ is the energy of the adsorbate in the 15 Å × 15 Å × 15 Å vacuum box, and *E*_(Surface+Adsorbate)_ is the energy of the surface interacting with the adsorbate.
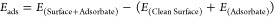
1

### 2.2 Adsorption–Regeneration Tests

**Figure 1:**
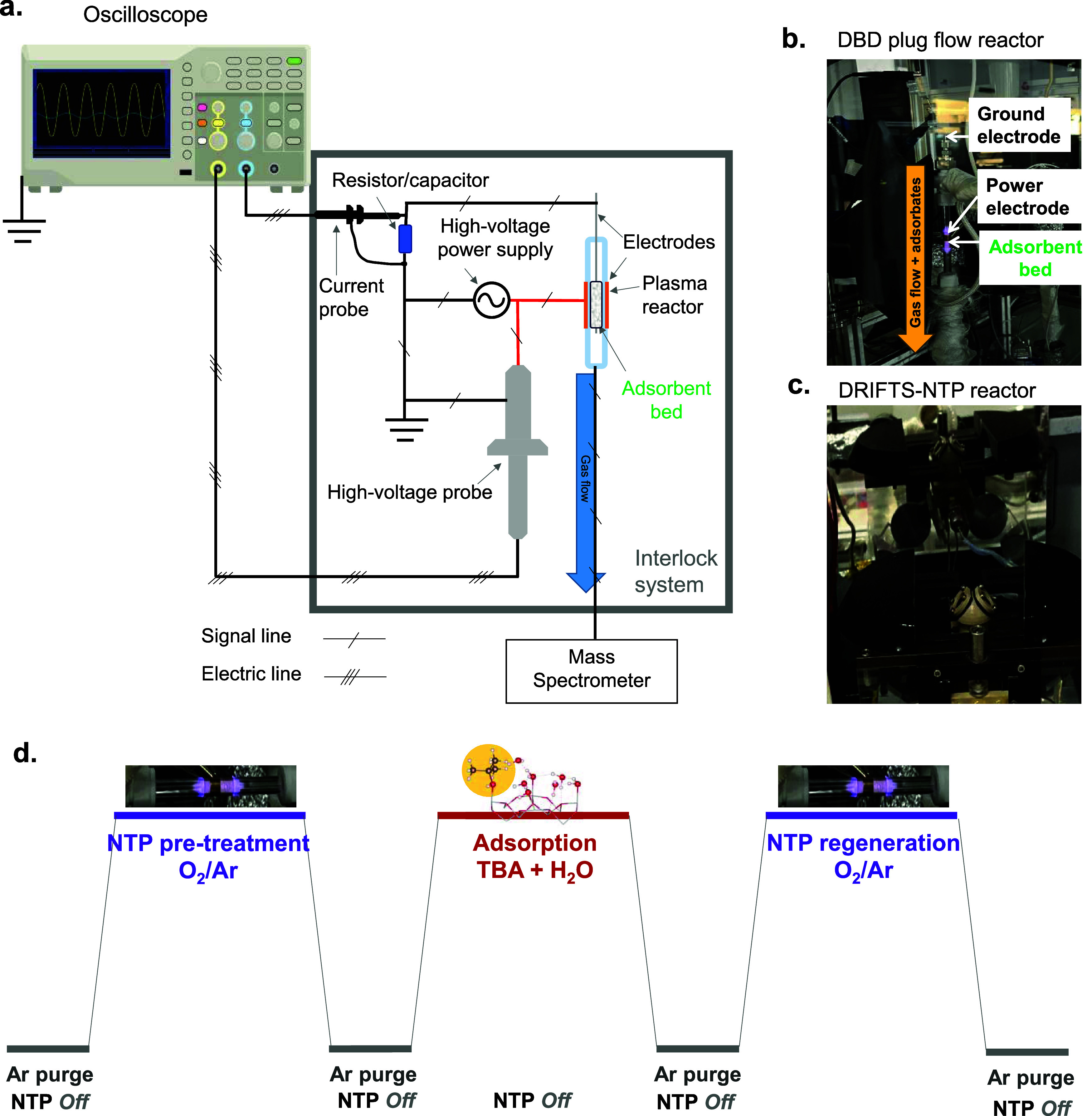
Experimental setup. (a) Schematics of the DBD reactor; (b) photograph of DBD reactor used for adsorption–regeneration tests; (c) photograph of plasma DRIFTS reactor used for mechanistic studies; and (d) schematic of the experimental protocol used during adsorption/regeneration studies of TBA over Al_2_O_3_ and TiO_2_ with NTP.

Typically, 25 mg of adsorbent was loaded between quartz wool plugs into a 6 mm OD quartz tube. A double dielectric barrier discharge (DBD) reactor was used for the treatment of the adsorbents, with a quartz-enclosed internal tungsten wire (0.5 mm OD) acting as a ground electrode and a metallic mesh on the outside of the reactor, used as a power electrode, as described elsewhere.^30,68^ Schematics of the setup and photographs of the reactors used for this study are shown in Figure 1.

The support was pretreated using the nonthermal plasma (NTP) for 30 min under a gas flow of 10 vol % O_2_/ 0.5 vol % Kr/Ar at 100 mL min^–1^ under ambient conditions to remove any adsorbed carbonaceous species. This was followed by a purge step under pure Ar at room temperature at 100 mL min^–1^ with the plasma off. During the adsorption step at room temperature (RT), the gas feed contained 2500 ppm TBA (as C4), 4% H_2_O (when added), and 0.5 vol % Kr in Ar, and the feed was left on for 30 min, to ensure the saturation point was reached. Another purge step followed the adsorption to allow the system to stabilize and then a regeneration step was carried out with 10 vol % O_2_/0.5 vol % Kr/Ar at 100 mL min^–1^ at ambient temperature under plasma (6 kV, 30 kHz, SIE 2.5–3 kJ L^–1^). Kr was used as an internal standard. The average power consumed during the plasma process and the specific input energy (SIE) were calculated as discussed in ref (68).

In order to monitor the changes in gas phase composition, the outlet of the reactor was connected to a Hiden Analytical HPR20 mass spectrometer via a heated capillary. The following mass-to-charge (*m*/*z*) ratios were monitored as a function of time: 18 (H_2_O), 32 (O_2_), 44 (CO_2_), 59 (TBA), and 84 (Kr). Quantification was carried out with reference to the Kr signal.

### 2.3 Mechanistic Studies

For insights into the adsorption–regeneration mechanisms of TBA over the Al_2_O_3_ support, *in-situ* DRIFTS studies were carried out. These used a previously reported DRIFTS reactor setup,^68^ and the same protocol as above was followed. The adsorbent material was placed in the ceramic crucible of the in-situ DRIFTS cell with its outlet connected to a Hiden Analytical HPR20 mass spectrometer via a heated capillary. The maximum applied voltage for the plasma tests was 5 kV. To compare the mechanistic studies during plasma treatment with conventional temperature-controlled adsorption–regeneration mechanisms, thermal tests have also been conducted. *In-situ* DRIFT spectra were recorded as a function of time during a temperature ramp of 10 °C min^–1^ from 30 to 300 °C. Prior to the reaction, a pretreatment under Ar was carried out at 300 °C, and data correction included the background subtraction.

The IR spectra were recorded with a resolution of 4 cm^–1^ and an accumulation of 128 scans every 60 s. The IR data is reported as log 1/*R* (“absorbance”), with *R* = *I*/*I*_0_, where *R* is the sample reflectance, *I* is the intensity measured under reaction conditions, and *I*_0_ is the intensity measured on the sample under a flow of argon. The *I*_0_ background spectrum was recorded at room temperature immediately prior to the introduction of the reactant mixture.

### 2.4 Liquid Phase Experiments

A test solution of 0.4 (v/v)% TBA was prepared in 18 MΩ deionized water, and another solution containing the same TBA concentration was prepared in tap water. A sample of the solutions was taken, and the analysis of the samples was carried out by gas chromatography (Agilent 7820A) using an HP-5 column with a flame ionization detector. Then, 5 mL of the two solutions were added into 10 mL brown vials, and 150 mg Al_2_O_3_ was added to each vial. The mixtures were stirred for 10 min and then allowed to reach equilibrium at room temperature for 3 days. A second liquid sample was taken from the vials containing the sorbent and analyzed by the GC to determine the adsorbed TBA using eq 2.
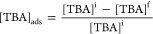
2where [TBA]_ads_ is the TBA adsorbed on Al_2_O_3_, expressed in μmol m^–2^, [TBA]^i^ is the initial concentration of TBA in the solution, and [TBA]^f^ is the final concentration of TBA in the solution at equilibrium.

## 3 Results and Discussion

### 3.1 DFT Investigation

DFT calculations for titania were performed using its most active phase (anatase) and the corresponding most stable surface (101), shown in the X-ray diffraction patterns (Figure S3) in the ESI. Due to the high complexity of the atomic structure of γ-alumina,^69^ DFT modeling was performed using the simpler α-Al_2_O_3_ (0001) surface. Despite the different phases, any trends computed for α-Al_2_O_3_ (0001) should be amplified in γ-alumina due to its higher surface area, Al density, and Lewis aciditiy.^70,71^

The absorbent characteristics of alumina and titania were initially explored by periodic DFT methods. These calculations were thus performed using slab models of the most stable surfaces of anatase and α-alumina: a-TiO_2_ (101) and α-Al_2_O_3_ (0001). The adsorption energies of geosmin and other model molecules (phenol, *tert*-butanol, and water itself) were initially computed over these surfaces. TBA was considered here as a simpler model system for geosmin.

For a-TiO_2_ (101), the calculated adsorption energy for phenol was −118.3 kJ mol^–1^ (Table 1). When geosmin and TBA were considered, the adsorption energies were only slightly more negative than that of phenol: – 144.1 and −120.9 kJ mol^–1^, respectively. The additional stabilization of geosmin compared to TBA is due to the noncovalent interactions between the surface and the alicyclic C–H bonds of this molecule. The calculated adsorption energy for the selected model molecules over the α-Al_2_O_3_ (0001) surface exhibited slightly more negative values than those for a-TiO_2_ (101). For geosmin and TBA, however, such differences were much more significant, reaching adsorption energy values of −202.1 and −153.1 kJ mol^–1^, respectively. Adsorption of H_2_O molecules resulted in calculated adsorption energies ranging between −111.2 and −86.2 kJ mol^–1^ over both a-TiO_2_ (101) and α-Al_2_O_3_ (0001) (Table 1), with only slight differences between the two surfaces. These preliminary results indicate that α-Al_2_O_3_ (0001) surface can bind geosmin and TBA molecules more strongly than a-TiO_2_ (101) surface, while the adsorption of other model molecules commonly found in wastewater showed similar adsorption energies in both cases.

**Table 1:** Calculated Adsorption Energies for Geosmin and Other Model Molecules Over a-TiO_2_ (101) and α-Al_2_O_3_ (0001) Surfaces

adsorbate	adsorption energy/kJ mol^–1^	
	a-TiO_2_ (101)	α-Al_2_O_3_ (0001)
phenol	–118.3	–137.6
TBA	–120.9	–153.1
geosmin	–144.1	–202.1
Water Adsorption		
1 H_2_O	–105.3	–111.2
2 H_2_O	–106.0	–101.6
3 H_2_O	–95.4	–106.4
4 H_2_O	–95.5	–86.2

**Figure 2:**
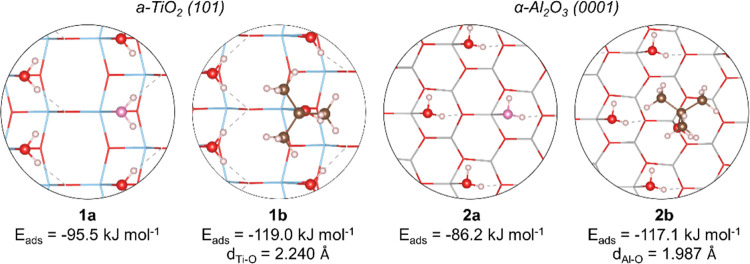
Adsorption of TBA and water over 3H_2_O/a-TiO_2_ (101) and 3H_2_O/α-Al_2_O_3_ (0001). Surfaces are represented by the stick model. Atoms are identified by colors: white (H), brown (C), red (O), light blue (Ti), and light gray (Al).

The effect of water was investigated further by the adsorption of TBA over partially hydrated surfaces. According to the surface model employed in this work, both a-TiO_2_ (101) and α-Al_2_O_3_ (0001) present four under-coordinated surface metal sites, which were considered for the adsorption of water. After partial hydration of these surfaces with three water molecules, the computed adsorption energies of *tert*-butanol were −119.0 and −117.1 kJ mol^–1^ for a-TiO_2_ (101) and α-Al_2_O_3_ (0001), respectively (1b and 2b, Figure 2). In comparison, the adsorption of a fourth water had a computed energy of −95.5 and −86.2 kJ mol^–1^ over a-TiO_2_ (101) and α-Al_2_O_3_ (0001), respectively (1a and 2a, Figure S1). Interestingly, the computed adsorption energy of TBA over a-TiO_2_ (101) was not significantly modified by the partially hydrated surface, whereas for α-Al_2_O_3_ (0001), a drop of 36.0 kJ mol^–1^ was computed due to steric repulsion between adsorbed waters and methyl groups of *tert*-butanol. As a result, the adsorption of TBA over both partially hydrated surfaces resulted in comparable adsorption energies.

**Figure 3:**
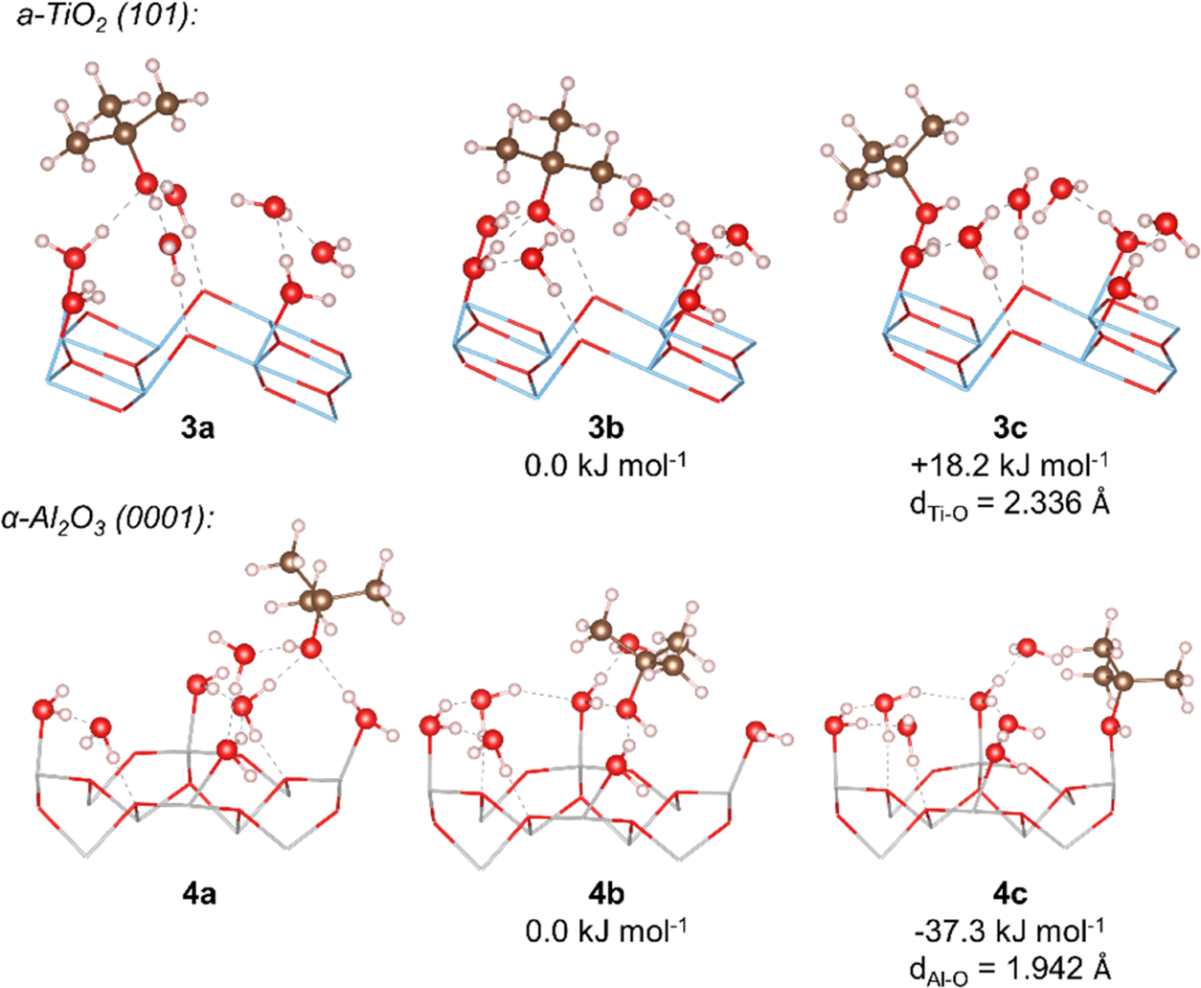
Adsorption stages and relative energies of microsolvated TBA over 4H_2_O/a-TiO_2_ (101) and α-Al_2_O_3_ (0001) surfaces. Surfaces are represented by the stick model. Atoms are identified by colors: white (H), brown (C), red (O), light blue (Ti), and light gray (Al).

In order to estimate the additional effect of the hydrogen bonding network formed by water molecules on these surfaces, the interaction between *tert*-butanol explicitly solvated by three water molecules (3H_2_O·TBA) and hydrated surfaces −4H_2_O/a-TiO_2_ (101) and 4H_2_O/α-Al_2_O_3_ was also considered. During the initial contact between these systems, the three water molecules initially solvating TBA migrated toward the surface to form a second solvation layer over the investigated surfaces (3a and 4a, Figure 3). However, the changes in energy caused by the interaction of the surfaces and the three additional water molecules are not relevant here, as these surfaces will be already fully solvated by water molecules in the real solution, and the additional stabilization computed here is simply due to the explicit solvation model. Nevertheless, the effect of water molecules over the interaction between TBA and the surface is still relevant for studying the competitive adsorption of water and *tert*-butanol. This effect was investigated using structures 3b and 4b (Figure 3), in which the hydroxyl group of *tert*-butanol forms two hydrogen bond interactions with adsorbed water molecules. From 3b and 4b, the positions of TBA and one water molecule in the first solvation layer were exchanged, leading to structures 3c and 4c and ensuring that the hydrogen bonding network formed over the surface is not significantly disturbed.

The exchange of one adsorbed water molecule by TBA over a-TiO_2_ (101) (3b → 3c) was found to be disfavored by +18.2 kJ mol^–1^. As can be seen in Figure 3, the Ti–O(TBA) bond length in the solvated system (2.336 Å) is larger than when considering the clean surface (2.200 Å) or a partially hydrated surface (2.240 Å; Figure 2). Additionally, if one additional hydrogen bond interaction is allowed to be formed between water and *tert*-butanol (Figure S2, Supporting Information), a further increase in energy of the system is computed (+9.2 kJ mol^–1^), and the Ti–O(TBA) bond length reaches 2.573 Å. These results show that strong hydrogen bond interactions between water and TBA will favor the desorption of this molecule from the surface. On the other hand, the exchange of one adsorbed water molecule by *tert*-butanol on α-Al_2_O_3_ (0001) is favored by −37.3 kJ mol^–1^. In this case, no significant changes in the Al-O(TBA) bond length were computed between explicitly solvated (1.942 Å) and clean surfaces (1.927 Å).

The preliminary evaluation of the adsorption of *tert*-butanol by DFT calculations revealed that TBA in an aqueous solution will only weakly interact with a-TiO_2_ (101), favoring the formation of hydrogen bond interactions with the water molecules on that surface instead. The opposite trend was computed on α-Al_2_O_3_ (0001) surfaces, in which adsorption of TBA leads to additional stabilization of the investigated system. These results suggest that alumina is the best candidate for capturing *tert*-butanol from an aqueous solution. The similarity with the values for geosmin also indicates a similar trend and that TBA is a reasonable choice as a model compound.

### 3.2 Adsorption–Regeneration Profiles

**Figure 4:**
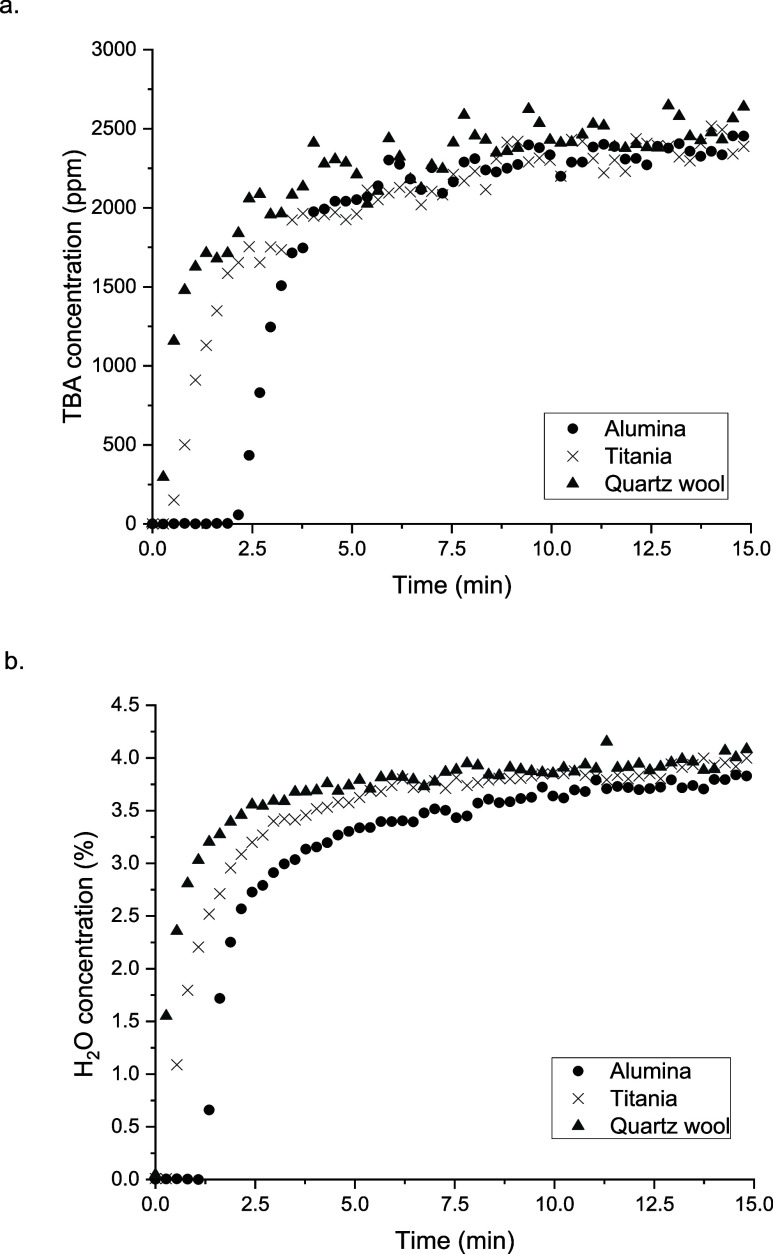
Adsorption profiles of (a) TBA and (b) H_2_O over Al_2_O_3_, TiO_2_, and quartz wool following NTP pretreatment; 2500 ppm TBA, 4% H_2_O/Ar, TFR = 100 mL min^–1^.

The adsorption profiles of TBA and H_2_O over alumina and titania are shown in Figure 4, with the results on the quartz wool alone, used conventionally to secure the packing of the supports, included for reference. The breakthrough of the TBA was observed within the first 15 s for the reactor containing only quartz wool and after additional 10 s for titania. The TBA breakthrough over the Al_2_O_3_ support occurred with a delay of 100 s compared to the blank reactor, as shown in Figure 4a. A similar trend was observed for the gas phase water, with breakthrough points after 5, 18, and 74s for the blank reactor, TiO_2_, and Al_2_O_3_, respectively. The quick breakthrough of water and sharp profile indicates insignificant adsorption on the blank reactor, as expected.

The short delay in the TBA breakthrough on TiO_2_ compared to the blank reactor indicates some adsorptive capacity of the TiO_2_ but lower than Al_2_O_3_, as reflected by the additional delay in the TBA breakthrough with alumina. The saturation point was reached on both of the sorbents within <30 min, as seen from the MS signals for the two sorbates (Figure S4, Supporting Information). The normalized data in terms of the amount of adsorbed TBA to the surface areas of the materials was also in line with the trends discussed, with 4.77 μmol m^–2^ TBA adsorbed on TiO_2_, compared to 6.11 μmol m^–2^ TBA adsorbed on Al_2_O_3_.

A competitive effect of water and TBA was observed over both supports investigated, as seen from the adsorption profiles (Figure S4, ESI), but it is worth noting that the presence of 4% H_2_O in the gas feed did not hinder the adsorption of the alcohol completely. However, the adsorption profiles indicate a distinct difference between the two supports, with titania displaying a lower adsorption capacity for the gas phase species present compared to alumina.

**Figure 5:**
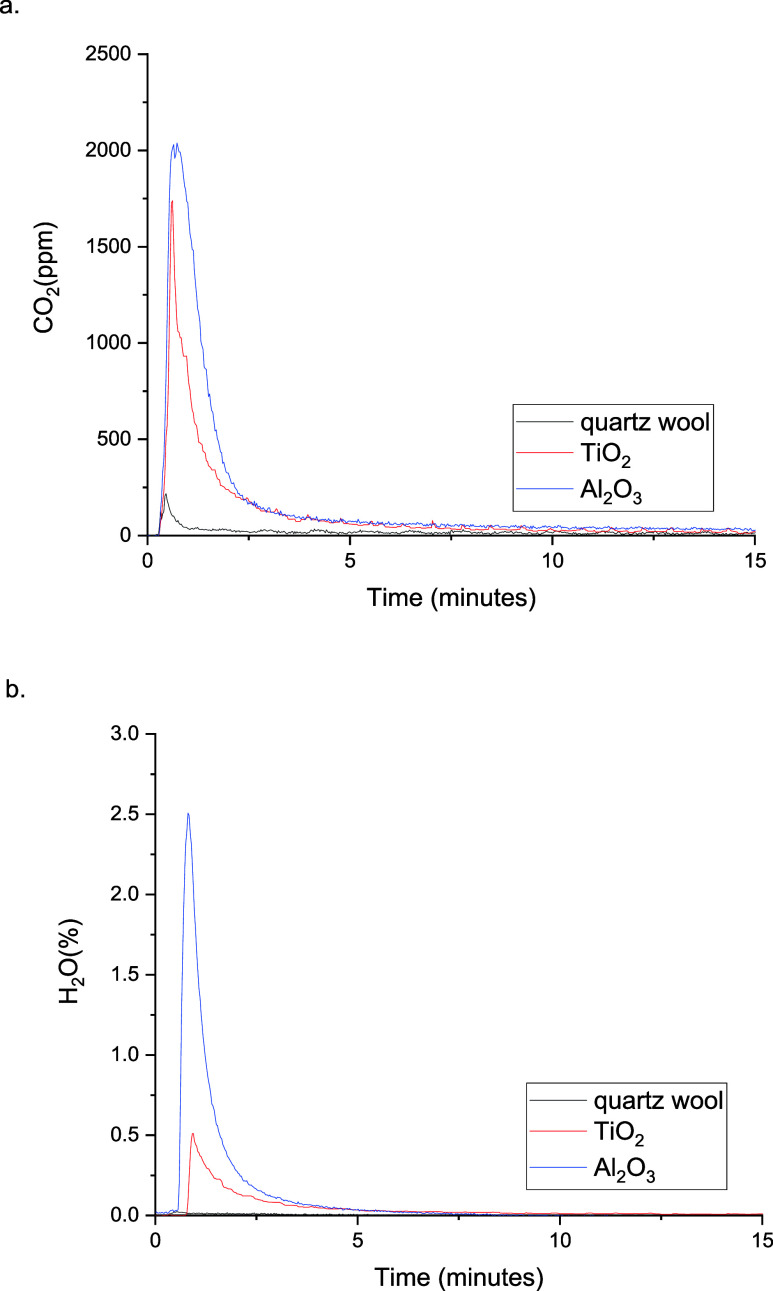
MS profiles of (a) CO_2_ and (b) H_2_O over Al_2_O_3_, TiO_2_, and quartz wool during the plasma regeneration step (6 kV, 27 kHz); 10% O_2_/Ar TFR = 100 mL min^–1^.

Following the adsorption step, the regeneration of the adsorbents was performed using an oxidizing feed (10% O_2_ in Ar) under DBD, at constant voltage and frequency corresponding to a power consumption of 4–5W and SIE 2.5–3 kJ L^–1^ (Figure S5, ESI). As shown in Figure 5, when the plasma was switched on, a sharp increase in the CO_2_ and H_2_O signals took place, indicating some mineralization of the surface adsorbents by the highly oxidizing species produced by the NTP. The concentration of the released species dropped considerably after approximately 5 min of plasma on and reached the baseline in less than 20 min. A small CO_2_ peak and a negligible water release were observed when the plasma was ignited over the blank reactor. The results of the regeneration test correlate well with the low adsorption profiles for TBA and H_2_O on quartz wool. A more significant increase in the CO_2_ signal took place during the plasma-assisted regeneration of TiO_2_ with 7.82 μmol of CO_2_ released over the 30 min plasma, compared to 1.63 μmol of CO_2_ from the quartz wool alone. However, the total amount of released CO_2_ from TiO_2_ was ∼64% lower than the CO_2_ released from Al_2_O_3_ (12.8 μmol) regeneration with NTP. By considering the effect of the surface area of the different supports and subtracting the CO_2_ released from the quartz wool alone, the normalized data with respect to BET surface area indicates that 27% more CO_2_ was released during plasma regeneration step from Al_2_O_3_ (3.06 μmol m^–2^) than from the TiO_2_ (2.40 μmol m^–2^). The release of water from the adsorbents closely followed the CO_2_ formation, with almost 3 times more H_2_O being released from the alumina compared to titania.

**Figure 6:**
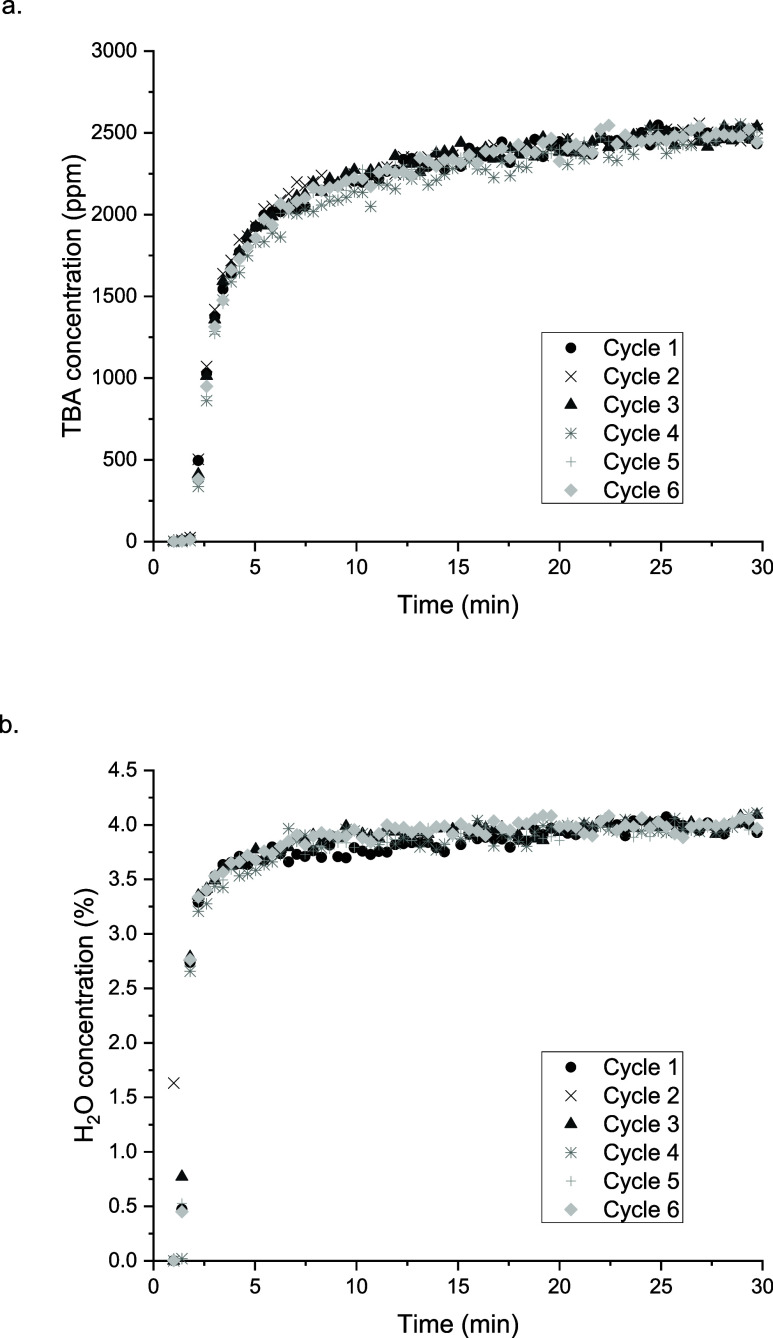
Regeneration of adsorption capacity for (a) TBA and (b) H_2_O over Al_2_O_3_, following NTP treatment; 2500 ppm of TBA, 4% H_2_O/Ar.

The adsorption/regeneration tests revealed that Al_2_O_3_ is likely to be a better candidate than TiO_2_ for adsorbing TBA from a wet feed, and this is in good agreement with the DFT findings. As a result, this indicates that photocatalysis may not be a suitable adsorption/treatment process for the removal of TBA from potable water under the conditions investigated. To assess the practical use of Al_2_O_3_ for this process, multiple adsorption–regeneration cycles using the same protocol were examined. The results of six consecutive cycles are shown in Figure 6. Insignificant differences in the breakthrough profiles for both TBA and water were observed over the 6 cycles, with TBA breakthrough occurring after approximately 2 min of exposure to the feed and reaching the saturation point after approximately 25 min. The regeneration of the support after each adsorption step was done as described above, using plasma and an oxidizing feed. The fast release of CO_2_ and H_2_O during the first 5 min of the plasma exposure and the good reproducibility of the TBA adsorption profiles suggest that alumina surface is easily cleaned up during the regeneration step, and subsequent adsorption/regeneration steps can be performed.

In order to have a better understanding of how the proposed adsorption/NTP regeneration system would perform in a practical system, the adsorption of TBA from liquid water was examined. A 0.4 (v/v)% TBA in deionized water (DI) was used, and alumina was examined as the sorbent material. The adsorbed TBA was calculated from the difference between the initial and final concentration of TBA in the solution using GC. Under these conditions, the TBA adsorbed on alumina from the liquid solution was 2.51 μmol m^–2^ compared to 6.11 μmol m^–2^ in the gas phase experiments. A further comparison was conducted using tap water instead of DI water using the same protocol in order to understand if other components in the water matrix (such as carbonates, phosphates, chlorides, and metal ions) affect the removal of TBA. It was found that the adsorption of TBA on Al_2_O_3_ increased when tap water was used compared with DI water, with 3.21 μmol m^–2^ TBA adsorbed. The observed 28% increase in adsorbed TBA from tap water is likely to be the result of complex interactions from the water matrix components with the adsorbent material. A similar effect on the decomposition of geosmin and MIB from raw river water vs distilled water was reported by Jo et al.^20^ and was attributed to the distinct differences in water quality, particularly due to the higher pH value of the raw river water matrix.

The tests indicate that the competitive effect between water and TBA adsorption on alumina is stronger in aqueous solutions than in a vaporised aqueous feed, given the ∼50% reduction in adsorbed TBA. However, the study is a proof of principle to show that the proposed 2-step system could successfully be used for the removal of low concentrations of contaminants from potable water using high surface area oxides. The sorbent materials can then be reused, following a regeneration step with NTP in an oxidizing environment, leading to mineralization of the impurities, without the need of additional steps for removal of byproduct contaminants as found for UV–H_2_O_2_ treatments of water containing MIB and geosmin.^72^

### 3.3 DRIFTS Analysis

The activity data indicate that under the conditions investigated, alumina presented the best adsorption properties for *tert*-butanol and similar regeneration behavior under plasma treatment. Further investigation of the adsorption of TBA over these supports was carried out using diffuse reflectance infrared Fourier transform spectroscopy (DRIFTS) using plasma and thermal treatment. The infrared cell for *in-situ* plasma measurements had been further developed to facilitate the investigation of different reaction environments and applied voltages under similar conditions with the plug flow reactor used for the gas phase adsorption profiles. The adsorption–regeneration protocol was kept the same for the plasma and the thermal tests, and the variations in surface species were monitored.

**Figure 7:**
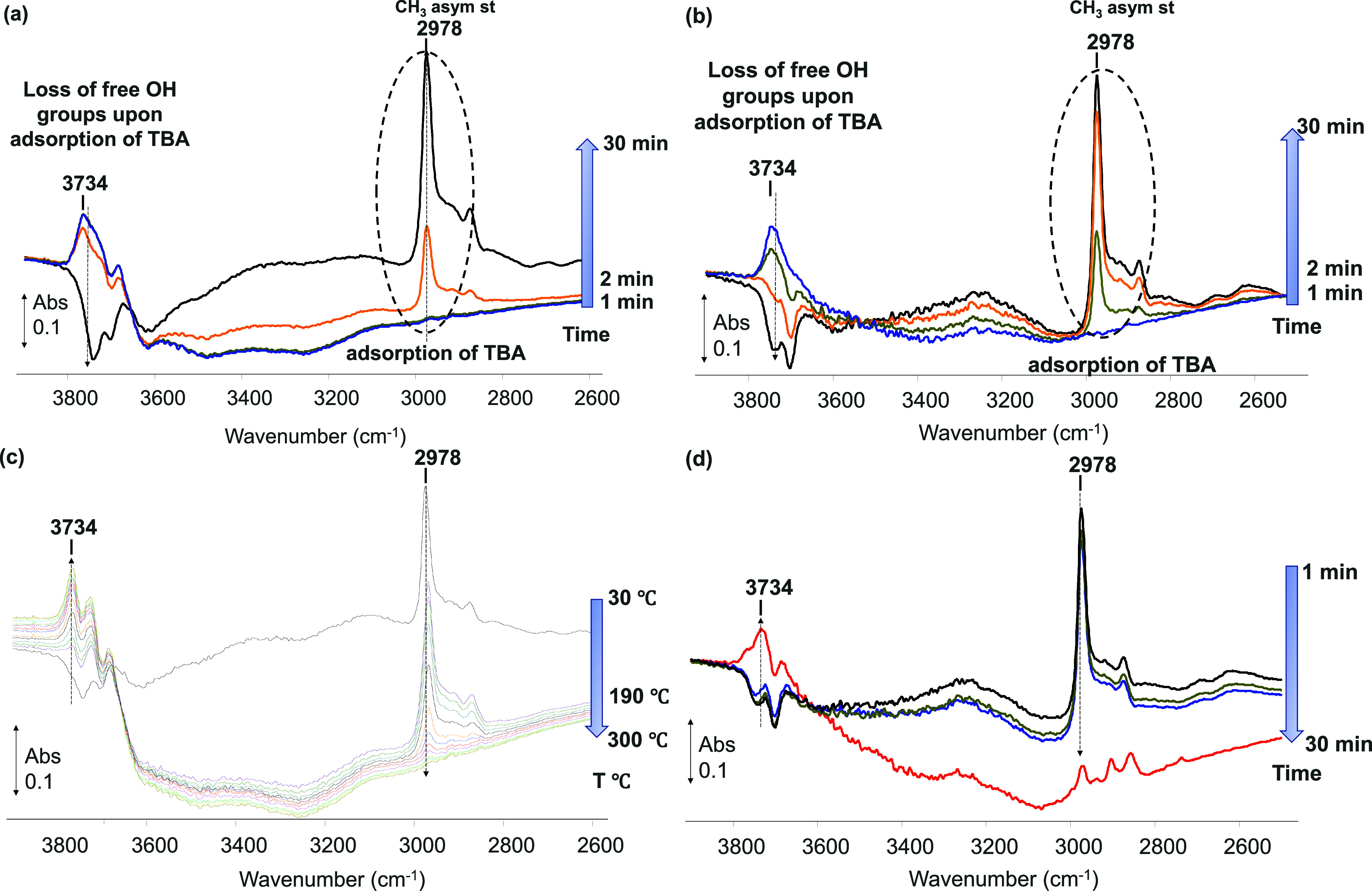
Absorption spectra recorded during the gas phase thermal adsorption (a)/removal (c) of *tert*-butanol; during the gas phase NTP adsorption (b)/removal (d) of *tert*-butanol from the alumina support (adsorption feed: 2500 ppm of TBA, Ar balance/removal feed: 10% O_2_, Ar balance, TFR = 100 mL min^–1^; 5 kV voltage, when applied).

The changes observed in the IR spectra during the adsorption step, as a function of time, are shown in Figures 7 a and b, with the TBA added, in the absence of water, following a pretreatment step at 300 °C or with NTP, respectively. A decrease in the band intensity at 3800–3590 cm^–1^ occurred once the adsorption feed was introduced to the support, and an increase in the bands at 3000–2800 cm^–1^ was observed, simultaneously. The negative bands at 3800–3590 cm^–1^ are associated with the OH-stretching vibration (ν_OH_),^68,73−75^ indicating the loss in terminal hydroxyl groups located at the surface of the alumina sample, while the observed increase in the bands at 3000–2800 cm^–1^ represents the CH_*x*_ asymmetric stretching of the *tert*-butanol molecule (ν_CHx_), indicating adsorption of the impurity on the support.

The IR spectrum of thermally activated γ-Al_2_O_3_ contained the same bands in the region between 3800 and 3590 cm^–1^, with the negative bands at 3734 and 3685 cm^–1^, assigned to hydroxyl groups on the surfaces.^74−76^ These bands diminished rapidly during the adsorption of TBA, as seen in the plasma experiments.

The regeneration step, with the temperature increasing from room temperature to 300 °C, shows a gradual decrease in the 2978 cm^–1^ peak area with temperature (Figures 7 c), while the changes in the negative shoulder from 3800 to 3590 cm^–1^ indicate the repopulation of the alumina surface with hydroxyl groups. No other changes have been observed in the spectra, which would indicate that the *tert*-butanol is desorbed from the alumina support thermally but requires temperatures of ∼250 °C to recover over 90% of its adsorption capacity.

**Figure 8:**
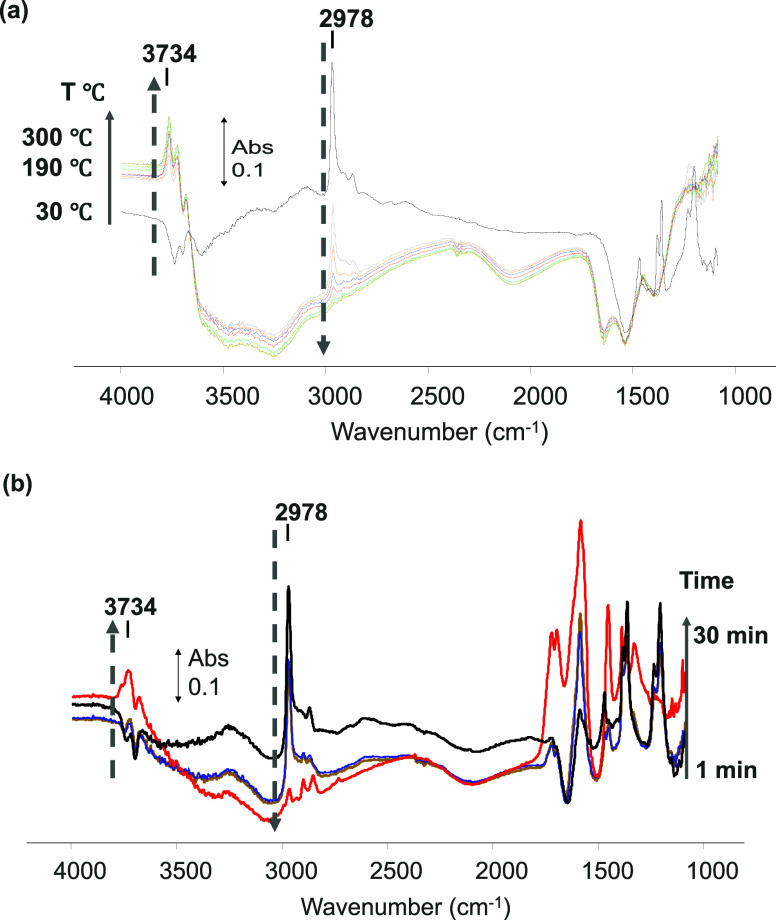
Evolution of the surface species on Al_2_O_3_ during the thermal (a) and plasma treatment at ambient temperature (b) (adsorption feed: 2500 ppm TBA, Ar balance/removal feed: 10% O_2_, Ar balance, TFR = 100 mL min^–1^; 5 kV voltage, when applied).

While the removal of TBA with conventional heating seems to be purely a gradual desorption process as a function of temperature, the NTP-assisted process involves a reactive mechanism. During plasma treatment, a change in the adsorbed species was clearly observed once the voltage was applied to the system. The presence of NTP and the oxidizing species created within the discharge interact with the adsorbed TBA molecule, leading to the formation of formate, carbonate, and carboxylate-type species, as shown in Figure 8. The IR bands at 2910 and 1589 cm^–1^ are ascribed to the stretching vibration ν_C–H_ and ν_OCO,_ respectively, derived from formate^76−78^ and also confirmed by the bending vibration (δ_C–H_) observed at 1393 and 1376 cm^–1^^74,77,78^ In line with previously reported data for oxidation of methane over alumina in the presence of plasma, an increase in the bands associated with formation of formates and carbonates (1700, 1646, 1459, and 1336 cm^–1^) was observed as soon as the voltage was set to 5 kV. The species formed during the plasma-step at 5 kV cannot be fully removed from the surface of alumina, and this is in good correlation with previously reported data on other oxidation reactions using plasma-catalysis systems.^68,79^ Among the effects of plasma discharge in the hybrid plasma-catalysis systems, a major role is attributed to the cleaning of the surface, but this can only be completed at higher voltages (≥6 kV) and/or with the aid of active metals. However, as in the case of thermal desorption step, the reduction in the TBA peak and the subsequent recovery of the free terminal OH- bands (3734 cm^–1^) are clearly seen on the DRIFT spectra (Figure 6) for the voltages investigated.

**Figure 9:**
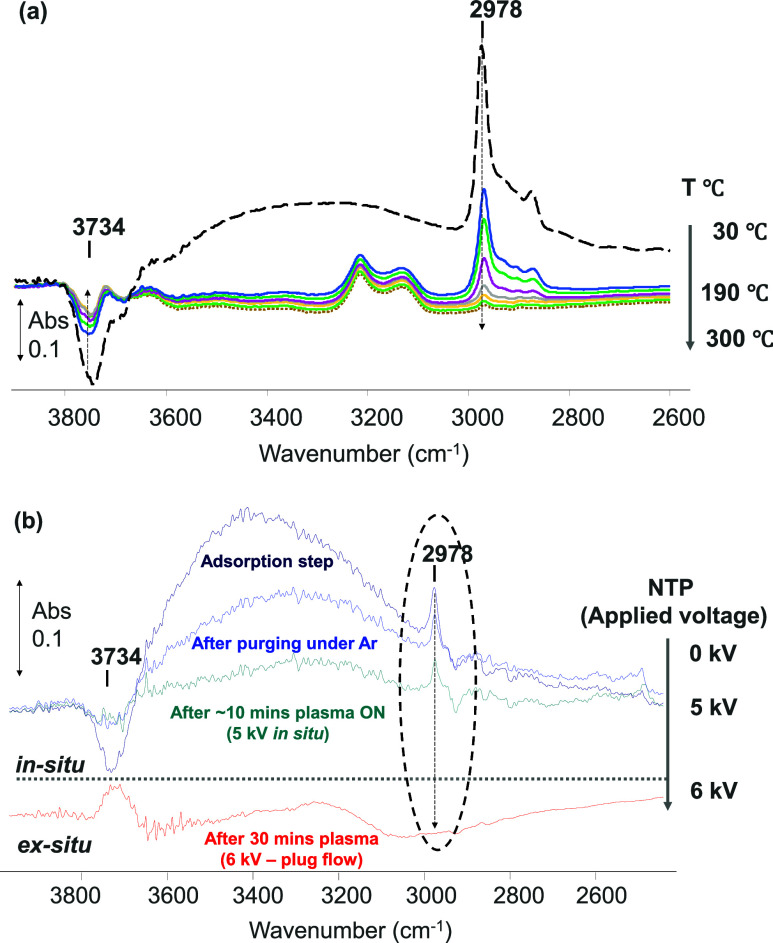
Evolution of the surface species on Al_2_O_3_ during the thermal (a) and plasma treatment at ambient temperature (b) (adsorption feed: 2500 ppm of TBA, 4% H_2_O, Ar balance/removal feed: 10% O_2_, Ar balance, TFR = 100 mL min^–1^; 5–6 kV voltage, when applied).

The competitive adsorption of TBA and water was also investigated in the reported conditions, and the representative spectra can be seen in Figure 9. The growth of the broad absorptions between 3600 to 1800 cm^–1^ is assigned to adsorbed water molecules. As the temperature increases, some of the weakly held water is quickly desorbed from the Al_2_O_3_ surface. However, the removal of TBA occurs more gradually and requires temperatures between 200–300 °C (Figure 9 a) to be fully desorbed from the support, with the subsequent recovery of the free −OH groups observed.

For the NTP experiments, a gradual increase in voltage was attempted with a reduction of <25% in the 2978 cm^–1^ peak area at 5 kV applied voltage (Figure 9b). Further increase in the voltage, with water present, resulted in arc formation; therefore, the sample analysis could not be carried out *in-situ* until complete removal of TBA from the alumina surface would occur. However, *ex-situ* experiments using the plug flow reactor were performed, and the sample was then analyzed using IR. As shown in Figure 9b, with the increase in voltage, the surface of Al_2_O_3_ was regenerated, the *tert*-butanol was removed, as well as the subsequent formate, carbonate, and carboxylate species observed at lower voltages.

**Figure 10:**
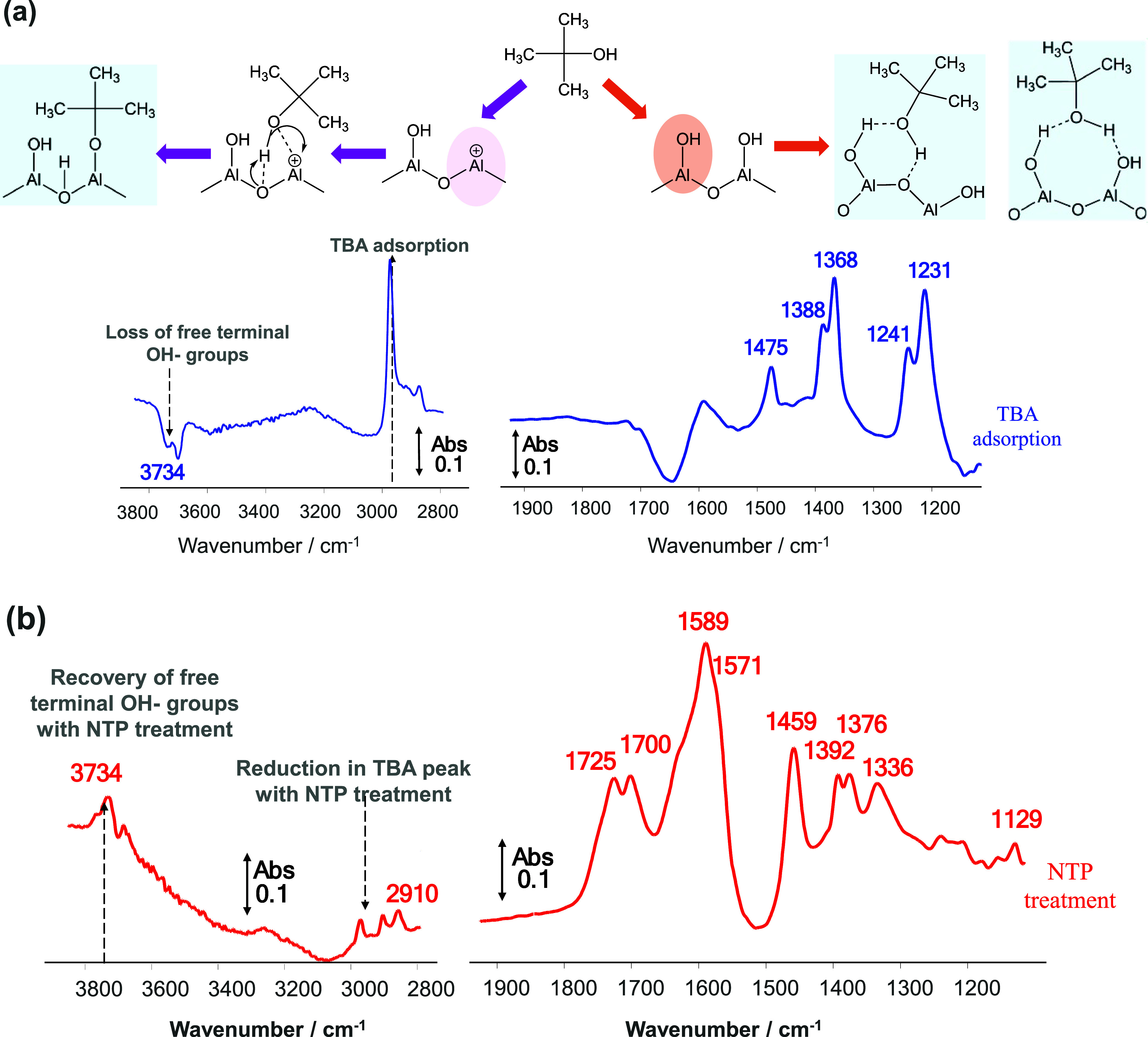
*In-situ* DRIFT spectra at 5 kV during (a) TBA adsorption and (b) regeneration of Al_2_O_3_ using NTP treatment.

**Figure 11:**
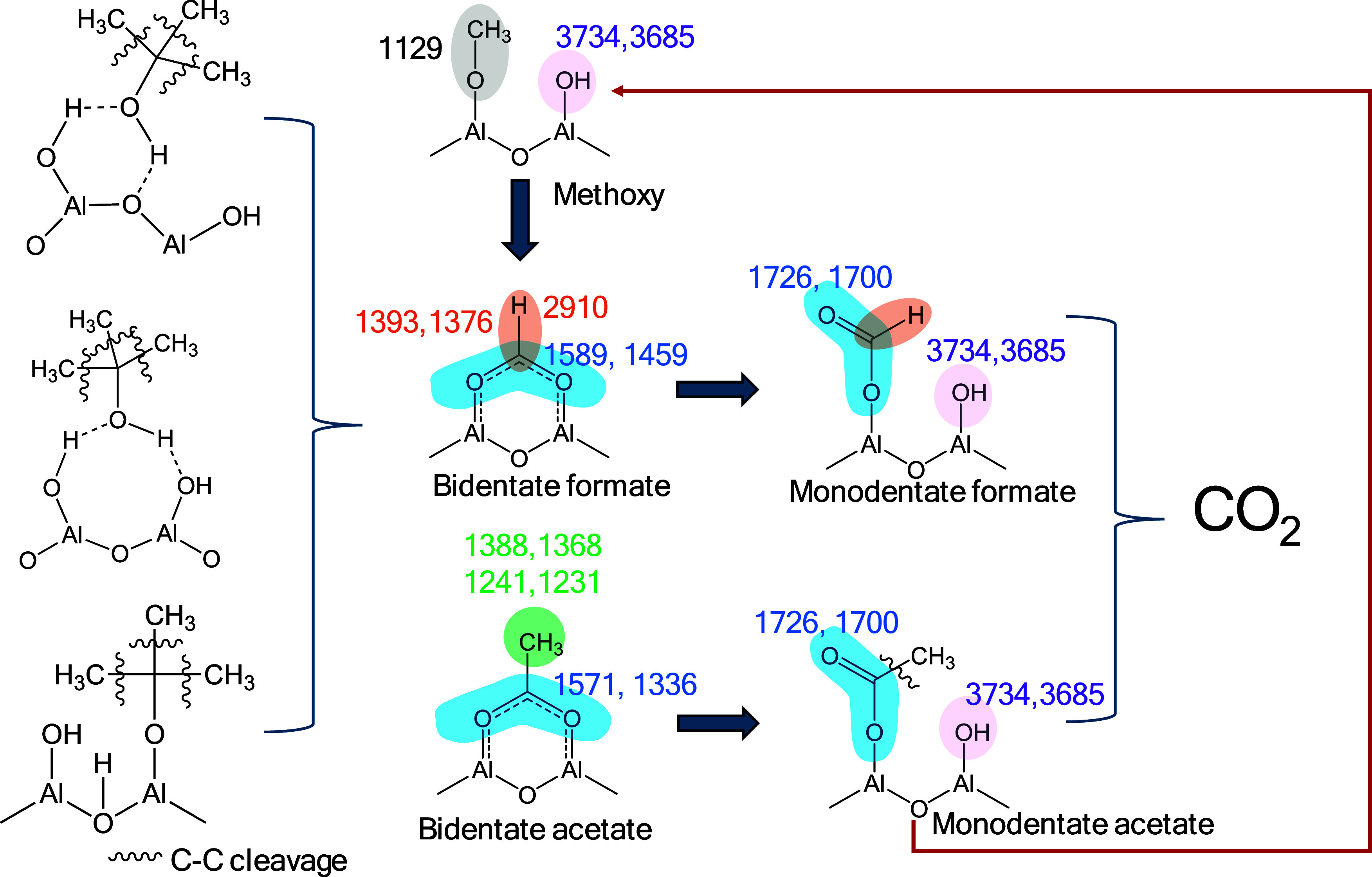
Proposed pathway for the plasma-catalytic regeneration process and reaction intermediates.

The plasma-catalytic regeneration involves chemical reactions as a result of the highly energetic species (e.g., electrons, metastables) and reactive oxygen species (ROS) generated in the DBD. These ROS have been proposed as an alternative to other methods of VOC removal from water matrices.^10,14,15,19,20,80−82^ In the current study, a comparison with thermal treatment of the adsorbents was made (Figure 9a), which mainly consists of the release of the impurities back into the environment. On the other hand, the O_2_–NTP regeneration step took place via a reactive mechanism between gas phase ROS (atomic oxygen, hydroxyl radicals, ozone, and radical ions, namely O_2_^+.^, O_2_^–.^, and O_3_^–^) and the adsorbents and adsorbate molecules. Thus, Figure 10 shows the comparison of typical *in-situ* DRIFT spectra obtained under TBA adsorption at room temperature (Figure 10a) and the O_2_–NTP treatment at 5 kV over the alumina sample. Significant differences were observed between the adsorption and regeneration spectra, in which several adsorbed species were detected (Figures 10, 11). As already discussed, the *in-situ* DRIFT spectra have significant features in the following IR regions: 3800–3590 cm^–1^ for stretching vibration of −OH group (ν_OH_), 3000–2800 cm^–1^ for stretching vibration of CH_*x*_ groups (ν_CHx_), 1750–1700 cm^–1^ for stretching vibration of carbonyl group (ν_CO_), and 1600–1100 cm^–1^ for stretching vibration of carboxylate-type species (ν_asOCO_ and ν_sOCO_) and bending vibration of −CH_*x*_ groups. Under the TBA adsorption on alumina at RT, the majority of surface species were associated with TBA, probably via hydrogen bonding with oxygen of the hydroxy-group on alumina, which is well supported by the reduction of the peaks associated with terminal OH- groups in the region (3734 cm^–1^) during the adsorption step, coinciding with the increase in the TBA peak.

In contrast, when the DRIFT spectrum was considered under an O_2_–NTP at 5 kV, a substantial change in surface species was observed. Figure 10b shows a characteristic IR band at 2910 cm^–1^, ascribed to the stretching vibration of formate species ν_C–H_, and the recovery of the hydroxyl group (3734 and 3685 cm^–1^) of alumina, indicating that TBA was removed or converted to other surface species. The IR bands at 1725 and 1700 cm^–1^ in Figure 10b indicate the existence of carbonyl groups ν_C=O_ of possible ***monodentate*** carboxylate-type species. Additionally, the IR band in the region of 1600–1100 cm^–1^ is attributed to the asymmetric and symmetric stretching vibration of ***bidentate*** carboxylate-type surface species (ν_asOCO_ and ν_sOCO_), and the bending vibration of bending vibrations of CH_*x*_ groups derived from partially converted TBA molecules. Based on the *in-situ* DRIFT spectra, Figure 11 shows a schematic summary of the proposed surface species under O_2_–NTP treatment at 5 kV. At higher discharge power, these adsorbed species can undergo further reaction to produce CO_2_, which fits well with the previously published data on NTP-assisted methane oxidation using Al_2_O_3_-based catalysts.^79^ Furthermore, *ex-situ* spectra of the regenerated Al_2_O_3_ at 6 kV presented in Figure 9 are in good correlation with the proposed mechanism, following complete removal of TBA and regeneration of the alumina surface by regaining terminal hydroxy groups, essential for subsequent adsorption cycles.

It is, thus, important to note that this surface reaction is dependent on energy input from NTP. At lower applied voltages, the energy provided will be sufficient to activate TBA and O_2_ molecules to form surface intermediates (e.g., formate, carbonate, and carboxylate species). However, mineralization of the TBA (eq 3) from the adsorbent will only take place at higher discharge powers as already discussed.
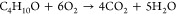
3

## 4 Conclusions

The combination of effective adsorbent materials that are often deployed in a variety of catalytic reactions together with the advanced oxidation process offered by nonthermal plasma gives an excellent platform for the development of a novel approach in potable water treatment. Computational data was used to identify possible supports for the adsorption of *tert*-butanol as a model for MIB and geosmin.

Both computational and experimental results show Al_2_O_3_ to be a superior absorbent to TiO_2_ for geosmin and MIB, giving non thermal plasma a potential advantage over photocatalysis for removal of these molecules. Consecutive adsorption–removal cycles were carried out without impacting the adsorption capacity of the alumina. The regeneration of the support following complete saturation was possible in plasma via the mineralization of the surface adsorbents.

The experiments under thermal conditions showed similar adsorption properties of the support but indicated that with the increase in temperature, the TBA is desorbed from the surface, with no other band changes observed, while the presence of plasma leads to oxidation of the impurity, with various intermediate/spectator species observed. DRIFTS data showed that the removal of TBA using plasma follows a reactive mechanism, with formates, carbonates, and carboxylate bands identified. However, the surface of the sorbent is regenerated, and removal of these intermediates was obtained with an increase in the applied voltage, which supports the findings from the subsequent adsorption–regeneration cycles, indicating that alumina is a suitable adsorbent for TBA and the adsorption capacity can be recovered quickly using a plasma-assisted treatment, with very good reproducibility between subsequent adsorption–regeneration steps.

These results indicate that this technology can be applied to other compounds and based on the computational studies, a possible molecule is geosmin. The development of a competitive method for removal of low-level impurities accountable for the unpleasant smell and taste of potable water is highly desirable and would be equally beneficial to society, industry, and the scientific community.

## Supporting Information Available

Additional computational and experimental data, including evolution of power consumption and SIE over time and XRD spectra for Al_2_O_3_ and TiO_2_. The data supporting the findings reported in this paper are openly available from the University of Manchester repository at https://figshare.manchester.ac.uk/ (PDF)
