# Supplementary material for: Removal and Oxidation of Low Concentration tert-Butanol from Potable Water using Nonthermal Plasma Coupled with Metal Oxide Adsorption
Source: ACS ES T Eng. 2024 Aug 20;4(9):2121–34. doi: 10.1021/acsestengg.4c00166 (PMC11406536; doi:10.1021/acsestengg.4c00166)
Supplement: Supplementary file 1 — ee4c00166_si_001.pdf [file ee4c00166_si_001.pdf]

Electronic Supporting Information

Removal and oxidation of low concentration *tert*-butanol from potable water using non-thermal plasma coupled with metal oxide adsorption

Cristina E. Stere[a], Maicon Delarmelina[b], Mbongiseni W. Dlamini[c], Sarayute Chansai[a], Philip R. Davies[c], Graham J. Hutchings[c] , C. Richard A. Catlow[b,d] and Christopher Hardacre[a]

[a]Department of Chemical Engineering, University of Manchester, Manchester, M13 9PL, UK

[b]Cardiff Catalysis Insitute, School of Chemistry, Cardiff University, Cardiff, CF10 3AT, UK

[c]Max Planck-Cardiff Centre on the Fundamentals of Heterogeneous Catalysis FUNCAT, Cardiff Catalysis Institute, School of Chemistry, Cardiff University, Main Building, Park Place, Cardiff, UK

[d]Department of Chemistry, University College London, 20 Gordon St., London WC1 HOAJ, UK

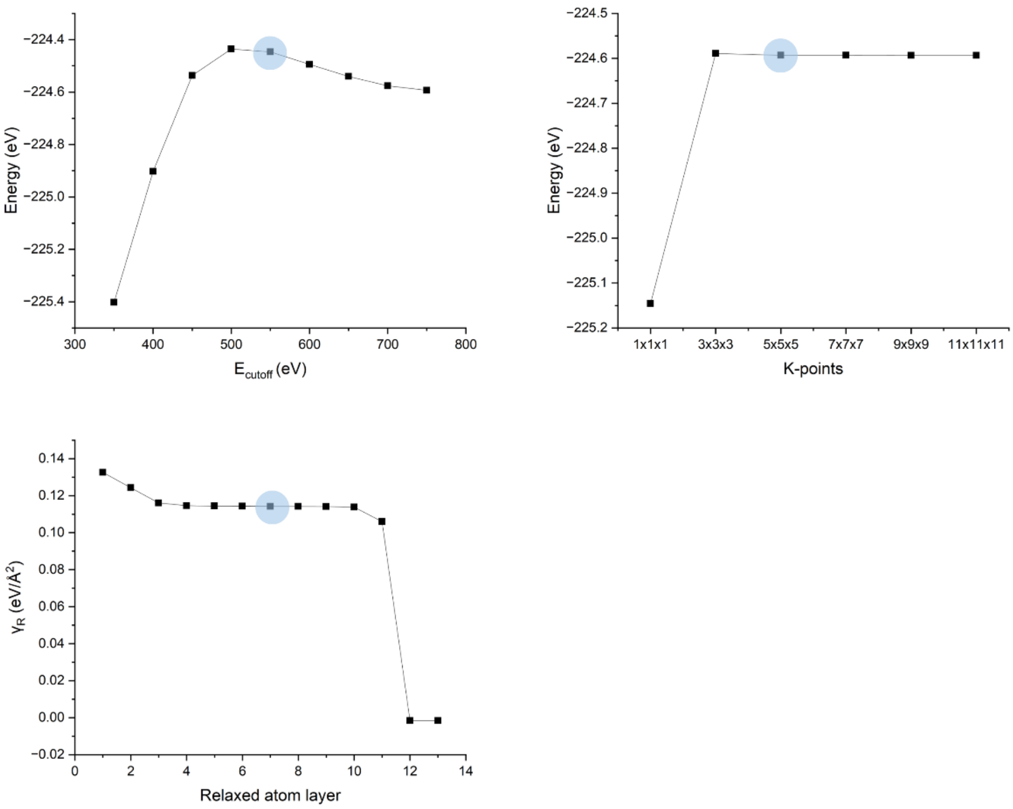

Figure S1. Preliminary investigation of the most appropriate K-point mesh and cutoff energies for modeling bulk α-Al_2O_3, and, required relaxed atom layers for the construction of the α-Al_2O_3(0001) slab model. Highlighted graph points indicate selected parameters.

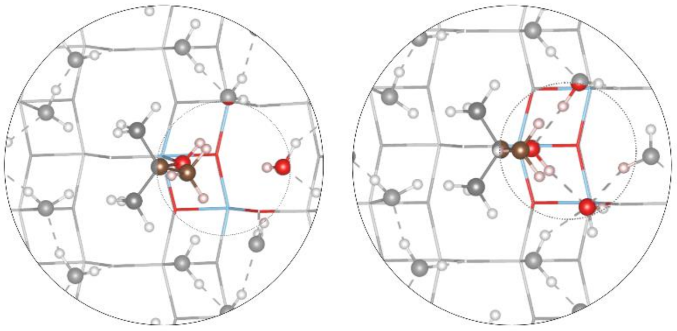

3c
E_{rel} = 0.0 kJ mol^{-1}
d_{Ti-O} = 2.336 Å

3d
E_{rel} = +9.2 kJ mol^{-1}
d_{Ti-O} = 2.573 Å

Figure S2. Effect of one additional hydrogen bond interaction over the adsorption energy of microsolvated TBA on 4H_2O/a-TiO_2.

The XRD patterns of titania and alumina shown in figure S3 were obtained by PANalytical X'Pert Pro X-ray diffractometer using CuKα emission lines from a generator operating at 40 keV and 40 mA. The XRD scans were performed at 2θ = 10°–90° with a step size of 0.033°. Analysis of the observed peaks revealed a mixture of phases in titania, corresponding to anatase (A): (101), (004), (200) and (211) planes and rutile (R): (110), (211) and (200) planes[1]. The X-ray diffraction pattern of Al_2O_3 shows several broad characteristic peaks at 2θ = 37.6º, 39.2º, 45.7º and 67.3º, corresponding to (311), (222), (400) and (440) planes[2].

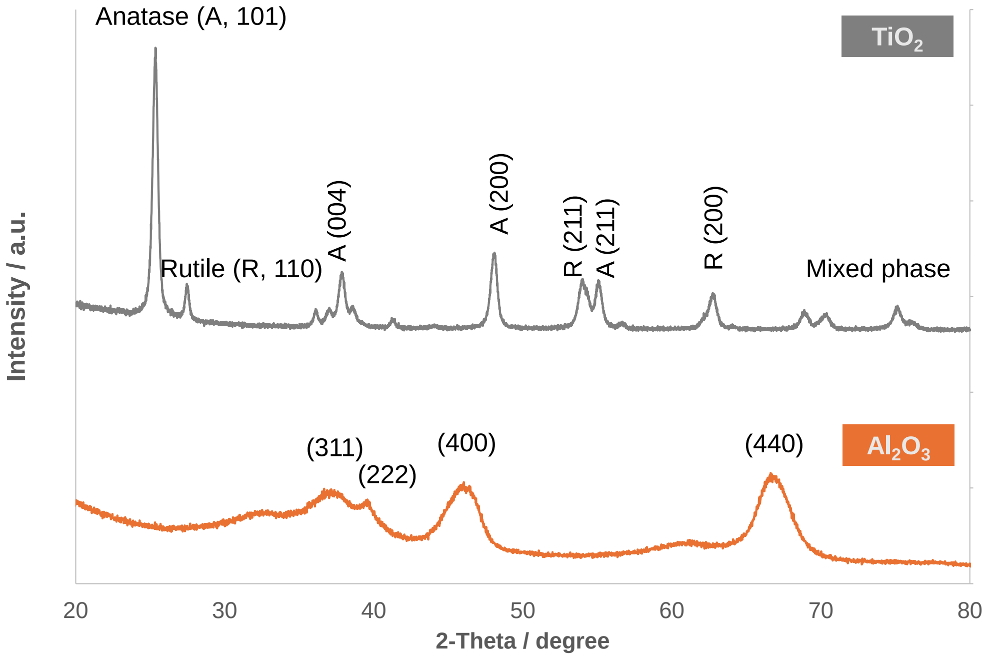

Figure S3. X-ray diffraction patterns of TiO_2 and Al_2O_3 supports.

a.

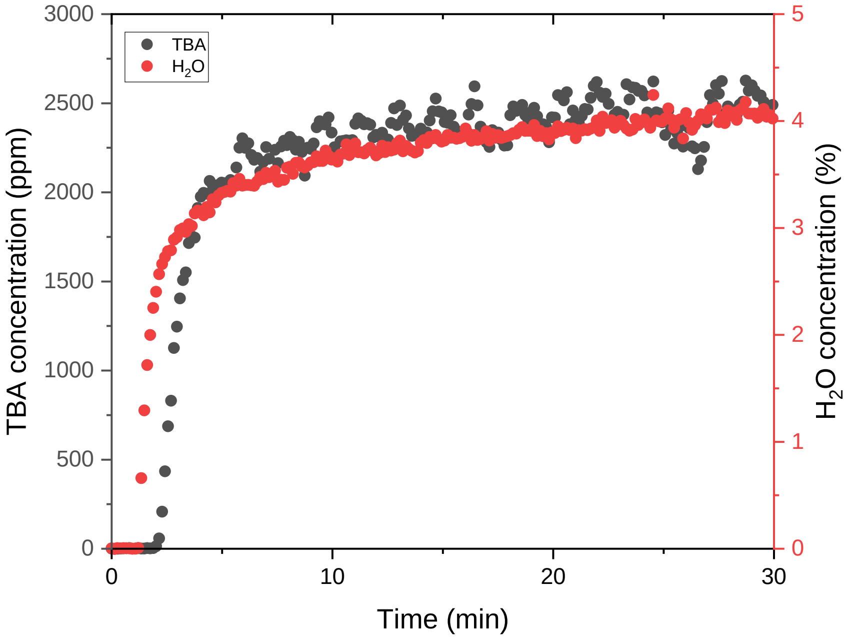

b.

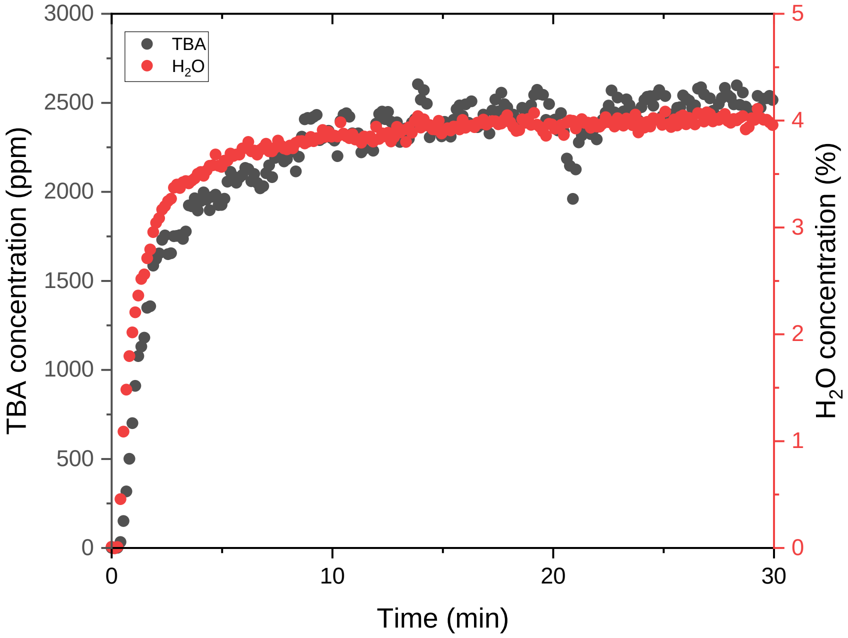

Figure S4. The TBA *vs* H_2O profiles during the adsorption step over (a) Al_2O_3 and (b) TiO_2; TFR = 100 mL min^{-1}, 2500 ppm TBA, 4% H_2O/Ar.

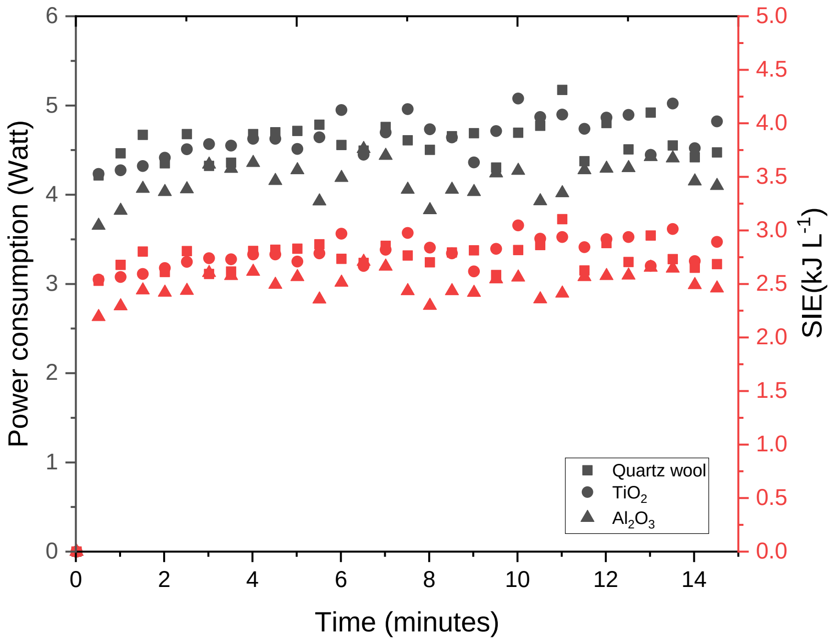

Figure S5. The variation of power (black) and SIE (red) over time during the regeneration step for quartz wool, TiO_2 and Al_2O_3; Experimental conditions: 10% O_2, Ar balance, TFR = 100 mL min^{-1}, plasma parameters: 6 kV, 27 kHz.

References

(1) Thamaphat, K.; Limsuwan, P.; Ngotawornchai, B. Phase Characterization of TiO_2 Powder by XRD and TEM. *Nat. Sci.* **2008**, *42* (5), 357–361.

(2) Kanwal, F.; Batool, A.; Adnan, M.; Naseem, S. The Effect of Molecular Structure, Band Gap Energy and Morphology on the Dc Electrical Conductivity of Polyaniline/Aluminium Oxide Composites. *Mater. Res. Innov.* **2015**, *19* (sup8), S8-354 - S8-358.
